# Perimenopausal giant hydatidiform mole complicated with preeclampsia and hyperthyroidism: A case report and literature review

**DOI:** 10.1515/med-2021-0315

**Published:** 2021-07-10

**Authors:** Yan Wan, Guoqing Jiang, Ying Jin, Zengping Hao

**Affiliations:** Department of Obstetrics and Gynecology, Beijing Friendship Hospital, Capital Medical University, Beijing 100050, China; Department of Obstetrics and Gynecology, The First Affiliated Hospital of Tsinghua University, Beijing 100016, China

**Keywords:** giant hydatidiform mole, perimenopausal, preeclampsia, hyperthyroidism

## Abstract

Gestational trophoblastic disease (GTD) commonly occurs in reproductive females, but is extremely rare in perimenopausal females. In this study, we reported a case of hydatidiform mole in a 48-year-old perimenopausal female admitted due to a giant uterine mass of 28 weeks’ gestational size. The serum human chorionic gonadotropin (HCG) level ranged from 944 to 1,286 mIU/mL before treatments. The signs of preeclampsia and hyperthyroidism were relatively prominent. Hysterectomy was performed and chemotherapy was scheduled when the serum HCG level remained at a plateau, about 528 mIU/mL. The symptoms of preeclampsia and hyperthyroidism were relieved after treatment. Accordingly, we concluded that GTD could occur in perimenopausal woman and hysterectomy usually is the optimal treatment.

## 1 Introduction

Gestational trophoblastic disease (GTD) is a group of diseases occurring in placental trophoblastic cells, which is characterized by excessive proliferation of placental trophoblastic cells and excessive villus edema [1]. It occurs mostly in reproductive women, but it has a low incidence in perimenopausal women [1]. The incidence of malignant degeneration is increasingly observed in perimenopausal patients with GTD [1]. In this paper, we reported a case of perimenopausal giant hydatidiform mole characterized by giant uterine lesions, signs of preeclampsia and hyperthyroidism.

## 2 Case presentation

**Figure 1:**
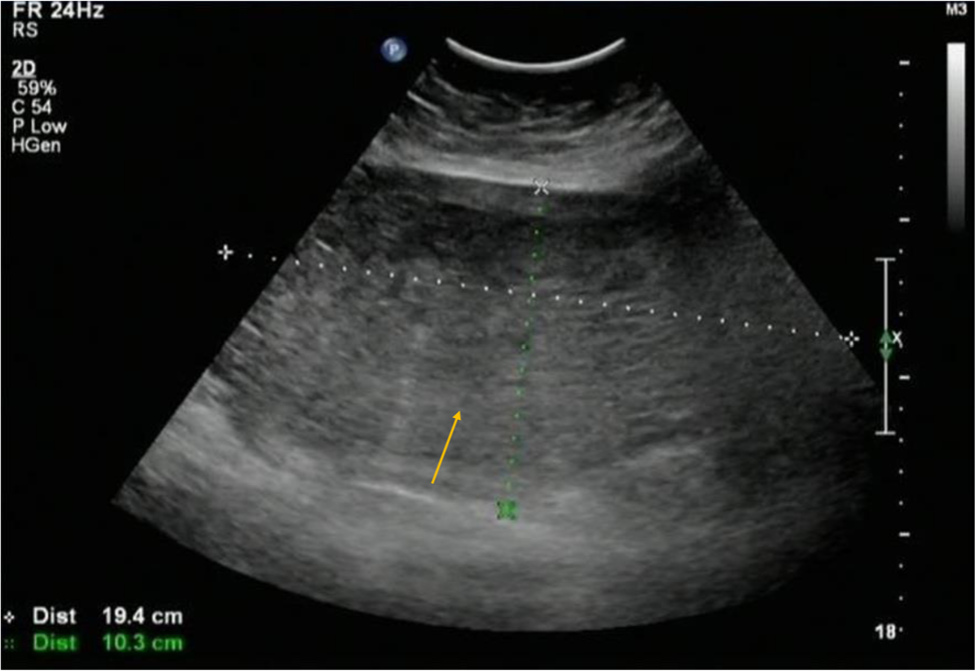
Ultrasound images showing a huge molar mass and faveolate subechoic area (arrow) in the uterine cavity.

**Figure 2:**
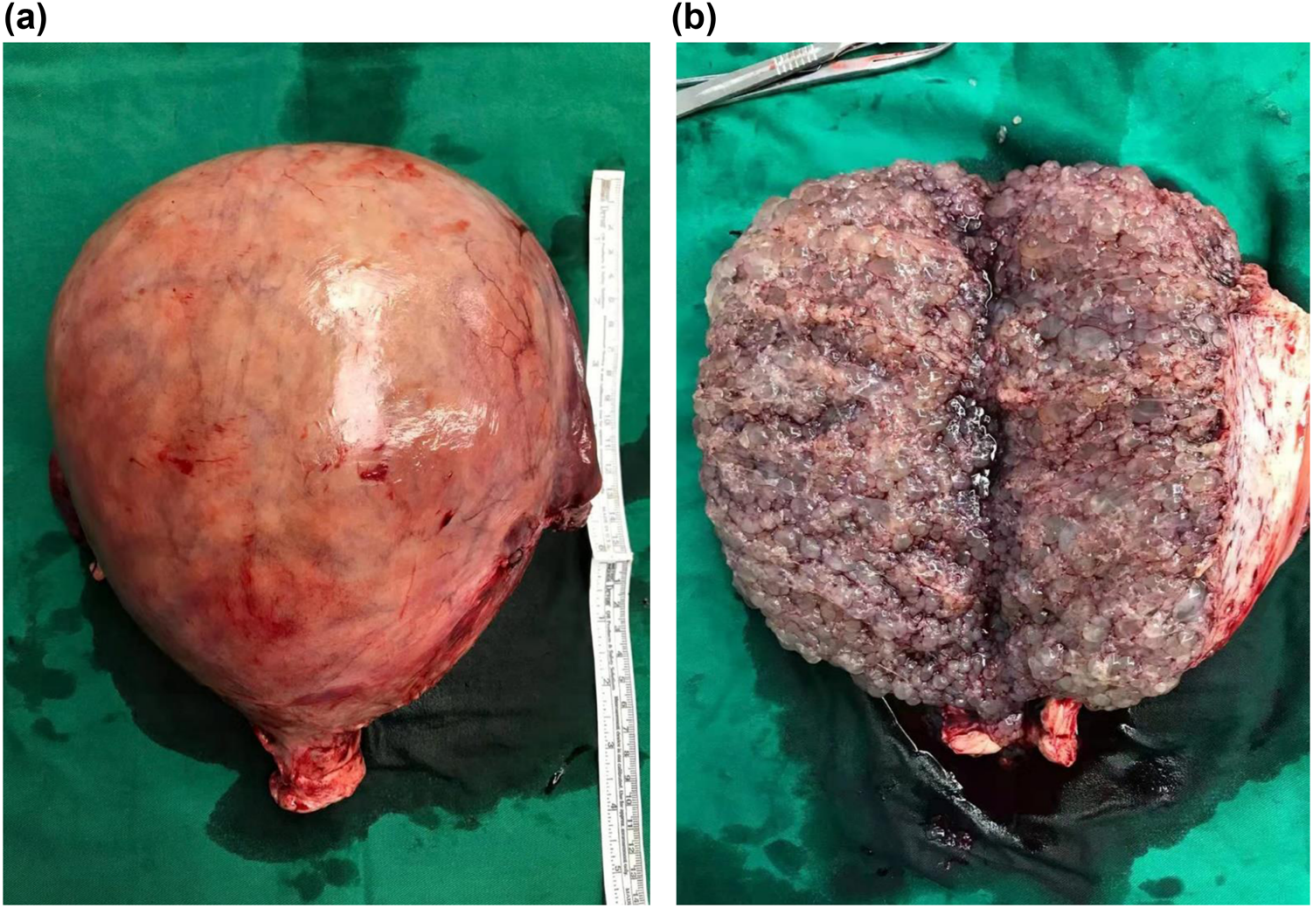
Macroscopic view of surgically removed uterine. (a) The size of the surgically removed uterine was 28 cm × 25 cm × 18 cm and weighed about 2,650 g. (b) The uterine cavity was filled with transparent grapelike tissue and the relationship with the muscular layer was still clear.


A 48-year-old female, gravida 6 para 4, was admitted due to vaginal bleeding lasting more than a month. Before admission, self-test of urine human chorionic gonadotropin (HCG) was negative twice. Since the onset of the disease, she had experienced intermittent nausea, dizziness, edema, significantly increased abdominal circumference, poor diet and sleep, and weight gain of 5 kg. After admission, blood pressure was 164/84 mm Hg and the heart rate was 100 beats/min; there was abdominal distension, with the distance from the bottom of the uterus to the umbilicus of 8 cm; there was no tenderness; there was edema of GRADE II; the HCG level was 944–1,286 mIU/mL; the TSH level was 0.01 uIU/mL with normal FT3 and FT4 levels; and the albumin level was 24.9 g/mL. The 24-hour urine protein was 3.58 g. Ultrasound showed that the uterine body size was about 19.4 cm × 16.9 cm × 10.3 cm. The faveolate subechoic area and a huge molar mass were seen in the uterine cavity (Figure 1) as well as a few blood flow signals, which suggested trophoblast disease. Echocardiography showed a slight enlargement of the left atrium. The preeclampsia signs (blood pressure, urine protein, edema, and hypoalbuminemia) and hyperthyroidism were also presented. However, the liver and kidney functions, cardiopulmonary function, and blood coagulation function were normal. The chest X-ray showed no nodules. The initial diagnosis was the presence of a hydatidiform mole complicated with preeclampsia and hyperthyroidism. In setting of administration of nifedipine (10 mg tid), furosemide diuresis (20 mg qd), methimazole (10 mg bid), albumin (10 g qd) supplementation and plasma (200 ml) infusion, no remission of symptom was observed but edema was aggravated (DEGREE IV). Therefore, after explaining the necessity of surgery to the patient and her relatives, total hysterectomy plus bilateral adnexectomy was performed. During surgery, about 500 mL of colorless ascites was seen. The size of the uterus increased as that of 28 week gestation, compressing other pelvic organs. The surface of the uterus was with edema filling with large blood vessels. The size of the uterus was 28 cm × 25 cm × 18 cm and weighed about 2,650 g (Figure 2a). Moreover, the uterine cavity was filled with transparent grapelike tissue, and the relationship with the muscular layer was still clear (Figure 2b). After surgery, she was transferred to intensive care unit. On the first day after surgery, the serum HCG was more than 269,800 mIU/mL. The blood pressure was normal on the third day after surgery. The thyroid function was normal on the postoperative 11th day; urine protein was negative on the postoperative 14th day; and edema was also obviously reduced. Histopathological examination also revealed a complete hydatidiform mole. The patient’s serum HCG level decreased to 528 mIU/mL at the postoperative 42nd day, entering a plateau period lasting for about 3 weeks. Additionally, the chest CT scan showed multiple nodules in both lungs at about 2 months after surgery. Thus, the invasive hydatidiform mole was diagnosed (stage III: seven points, high-risk group). According to the *Guidelines for Diagnosis and Treatment of GTD*s (Fourth Edition), the patient was given 5-fluorouracil + actinomycin D chemotherapy. After one course of treatment, the serum HCG level returned to a normal level, suggesting that the invasive hydatidiform mole is sensitive to chemotherapy. Then, three courses of consolidation chemotherapy were administered. Until last follow-up in November 2020, she recovered well, with a negative serum HCG, normal blood pressure, negative urine protein, and normal thyroid function. The chest CT and pelvic ultrasound showed no abnormal lesions.

**Ethical approval:** This case report was approved by the Ethics Committee of Beijing Friendship Hospital, Capital Medical University.
**Informed consent:** Informed consent has been obtained from patient included in this study.

## 3 Discussion

Giant hydatidiform mass could rarely be found in perimenopausal women. The clinical characteristics, pathogenesis, complications, and treatments of giant hydatidiform mass in perimenopausal females are significantly different from those of reproductive age. To date, a total of six cases of giant hydatidiform mole in perimenopausal women aged from 51 to 60 years have been reported in the available literature studies [2,3,4,5,6,7]. Among these six cases, two were complicated with hyperthyroidism, four underwent hysterectomy, one underwent hysterectomy and chemotherapy, and only one case was treated with suction curettage plus chemotherapy. The main features of these cases are summarized in Table 1. However, there have been no literature reports about the giant hydatidiform mole complicated with preeclampsia and hyperthyroidism in perimenopausal females.

**Table 1:** All six published cases of giant hydatidiform mole in postmenopausal woman

Year	Authors	Age (years)	Symptoms	Size of uterus	HCG (mIU/mL)	Treatment	Complication	Recurrence
1994	Pirhonen et al. [2]	56	Vaginal bleeding	16 weeks of gestation	>100,000	Hysterectomy	None	None
2005	Lok et al. [3]	56	Abdominal pain and vaginal bleeding	24 weeks of gestation	>100,000	Hysterectomy	Hyperthyreosis	None
2006	Taskin et al. [4]	53	Abdominal pain	16 weeks of gestation	>100,000	Hysterectomy	None	None
2009	Struthmann et al. [5]	53	Abdominal pain and vaginal bleeding	26 weeks of gestation	>1,000,000	Suction curettage + chemotherapy	Hyperthyreosis	None
2012	Mehrotra et al. [6]	60	Abdominal pain and vaginal bleeding	24 weeks of gestation	>200,000	Suction curettage + hysterectomy	None	None
2016	Akyol et al. [7]	51	Abdominal distension and vaginal bleeding	28 weeks of gestation	>200,000	Hysterectomy + chemotherapy	Invasive mole	None

Generally, 10^5^ active trophoblastic cells secrete 1 IU HCG every 24 h [8]. In this case report, a large number of trophoblastic cells were present in the giant uterine cavity of patients, which should result in a high level of serum HCG. However, her preoperative serum HCG levels were very low, ranging from 944 to 1,286 mIU/mL. There are differences in trophoblast proliferation and villous stromal edema between perimenopausal and reproductive women with hydatidiform moles [9]. In perimenopausal females with a mole, there may be a higher proportion of trophoblast necrosis, which may lead to a low level of secreted HCG. What needs to be considered here is the reason for the significant increase in HCG level after surgery: is it caused by the invasion of trophoblast cells into the blood during the operation? Or is it because the low level of HCG before surgery is the illusion caused by the “hook effect?” The “hook effect” is also known as “prozone phenomenon,” which may be another reason for a low-level HCG [10]. Yeung and Cheung reported that in setting serum levels of HCG above 500,000 mIU/mL, a “hook effect” could occur, resulting in an artificially low or negative value when using the current commercially available immunometric HCG assays [11]. A 1:10 to 1:1,000 dilution of the antigen sample may overcome the hook effect by reducing the concentration and allowing the antibodies to suitably bind to two portions of the same molecule [12]. Therefore, when high level of HCG is suspected, serum should be diluted before detection to avoid the hook effect. In this report, the preoperative specimens had not been retained and thus the reasons of this phenomenon are unclear. Further studies are warranted.

Preeclampsia is typically characterized by elevated blood pressure, positive urine protein and/or systemic damage after 20 weeks of gestation [13]. The basic pathophysiological changes are small vasospasm and vascular endothelial injury. For preeclampsia treatment, except lower blood pressure, sedatives, and diuresis, the most critical is the use of magnesium sulfate for the prevention of eclampsia [14]. The patient presented typical signs of preeclampsia: high blood pressure, positive urine protein, hypoproteinemia, and obvious edema. However, there were no obvious abnormalities in the assessment of various organs throughout the body, including cranial and fundus vascular lesions, liver and kidney functions, cardiopulmonary functions, and blood coagulation functions. Therefore, prophylactic use of magnesium sulfate for spasmolysis was not studied. In perimenopausal patients with signs of preeclampsia in hydatidiform mole, there are yet no guidelines, reports, or experiences on whether magnesium sulfate should be applied to prevent eclampsia, which needs to be further discussed.

Suction curettage is the preferred treatment for complete hydatidiform mole, especially for patients with fertility requirements [15]. However, total hysterectomy may be appropriate for perimenopausal patients who have already given birth. In addition to the removal of the hydatidiform tissue, total hysterectomy can reduce the need for subsequent chemotherapy by eliminating the risk of local muscular layer infiltration [16]. Zhao et al. [15] showed that the incidence of progression to GTN after suction curettage in high-risk hydatidiform mole patients above 40 years old was 44%. Total hysterectomy could reduce the possibility of GTN progression, but 13% of patients could still progress to GTN [16]. Thus, standard postoperative monitoring was still needed. In this study, there were a series of clinical features in this case, mainly the following: (1) The patient was a 48-year-old perimenopausal woman with a large uterus and positive serum HCG, with no intrauterine fetal tissue found by ultrasonography. (2) Serum HCG was detected at a low level, which was not consistent with the enlarged uterus. (3) The signs of preeclampsia and hyperthyroidism were relatively prominent. (4) The disease progressed rapidly. The risk of acute and severe complications was high, such as uterine rupture, uncontrolled massive hemorrhage, hyperthyroidism, and other diseases. After comprehensive evaluation, suction curettage and suction curettage plus chemotherapy were found to be not the optimal treatment for this patient; hence hysterectomy plus bilateral adnexectomy were performed finally. Due to the giant uterus as well as abundant blood vessels of uterine surface and periuterine tissues, the risk of massive bleeding and pelvic organ damage was high. Therefore, the operation was performed by experienced gynecologic surgeons. Preoperative blood preparation and intraoperative gentle operation were necessary. Both macroscopic evaluation and postoperative pathology showed a complete hydatidiform mole. After surgery, blood pressure quickly resumed to normal; urine protein gradually resumed to negative; edema was obviously alleviated; and thyroid function also resumed to a normal level. Thus, it can be seen that hysterectomy can be a primary treatment for women with non-metastatic GTD [1].

Elias et al. [17] selected 22 patients aged 50 or older who presented with a primary diagnosis of complete hydatidiform mole, and found that 60% of the 15 patients initially treated with dilation and curettage developed GTN, while there was no case of GTN among the seven patients treated with primary hysterectomy. They concluded that hysterectomy should be considered as the initial treatment for complete hydatidiform mole. Cagayan and Magallanes [18] showed that early surgical intervention combined with chemotherapy could reduce the number of chemotherapy cycles for remission and decrease the exposure of the patients to toxicity from chemotherapy. In this study, invasive hydatidiform mole was found after hysterectomy, which could not have been caused by the surgery itself, but associated with the high risk factors such as 48-year old, a giant uterine, preeclampsia, and hyperthyroidism.

Wang et al. [19] reported that preventive chemotherapy could indeed reduce the chance of complete hydatidiform mole progressing to GTN, and the effect was more obvious for patients with high-risk factors. However, some complete hydatidiform mole patients still progress to GTN after prophylactic chemotherapy, who have a greater possibility of drug resistance and need more courses of chemotherapy to achieve cure [9]. Therefore, whether complete hydatidiform mole patients need prophylactic chemotherapy is still a controversial topic at present.

In conclusion, the incidence of perimenopausal giant mole complicated with preeclampsia and hyperthyroidism is rare. The risk of progressing to GTN is significantly increased. Total hysterectomy is the main recommended treatment at present and close follow-up after surgery is essential; whether to undergo adjuvant chemotherapy is determined according to the conditions of patients.
